# Ce^3+^/Ce^4+^-Doped ZrO_2_/CuO Nanocomposite for Enhanced Photocatalytic Degradation of Methylene Blue under Visible Light

**DOI:** 10.3390/toxics10080463

**Published:** 2022-08-10

**Authors:** Manh Nhuong Chu, Lan T. H. Nguyen, Mai Xuan Truong, Tra Huong Do, Thi Tu Anh Duong, Loan T. T. Nguyen, Mai An Pham, Thi Kim Ngan Tran, Thi Cam Quyen Ngo, Van Huan Pham

**Affiliations:** 1 Faculty of Chemistry, Thai Nguyen University of Education, Thai Nguyen City 24000, Vietnam; 2 Faculty of Physics, Thai Nguyen University of Education, Thai Nguyen City 24000, Vietnam; 3 Institute of Applied Technology and Sustainable Development, Nguyen Tat Thanh University, Ho Chi Minh City 700000, Vietnam; 4 Advanced Institute of Science and Technology, Hanoi University of Science and Technology, Hanoi City 100000, Vietnam

**Keywords:** Ce^3+^/Ce^4+^, mixed semiconductor oxide, hydrothermal, photocatalysis, methylene blue

## Abstract

In recent years, photocatalysis has been used as an environmentally friendly method for the degradation of organic pigments in water. In this study, Ce^3+^/Ce^4+^-doped ZrO_2_/CuO as a mixed semiconductor oxide was successfully prepared by a one-step hydrothermal method. The Ce^3+^/Ce^4+^-doped ZrO_2_/CuO has shown high degradation efficiency of methylene blue (MB), and the maximum degradation percentage was observed to be 94.5% at 180 min under irradiation visible light. The photocatalytic activity increases significantly by doping Ce^3+^/Ce^4+^ in ZrO_2_/CuO for MB degradation. Ce^3+^/Ce^4+^ doping is shown to reduce the (e^-^/h^+^) recombination rate and improve the charge transfer, leading to enhanced photocatalytic activity of materials. The materials were characterized by X-ray diffraction (XRD), scanning electron microscopy (SEM), transmission electron microscopy (TEM), FTIR, EDS, BET and diffuse reflectance spectroscopy (DRS).

## 1. Introduction

Water pollution is a global environmental problem as it can lead to the decomposition of aquatic ecosystems and can affect human health. Contaminants can include organic or inorganic compounds, metal ions, dyes, phenols, pesticides and detergents. In which the pollution of natural and synthetic organic dyes poses challenges because of their high carcinogenic nature due to the fact they contain azo functional groups that, during decomposition, can form amines and benzidine [1]. Organic dyes are persistent organic pollutants that are resistant to decomposition through chemical and biological processes. The dye molecules are practically non-biodegradable, so they persist longer in the environment, creating potential hazards [2].

Today, water pollution is treated by applying technologies such as adsorption, coagulation, filtration and photocatalysis. In which the heterogeneous photocatalysis method has gained wide scientific attention with high potential for applications [3]. Zirconia (ZrO_2_)-based photocatalysis for wastewater treatment is of great interest because ZrO_2_ is a semiconductor oxide with high mechanical strength, is non-toxic, and has high chemical stability and biocompatibility. However, ZrO_2_ has a large band gap; the E_g_ of ZrO_2_ ranges from 3.25 to 5.1 eV, depending on the fabrication method [4]. Therefore, ZrO_2_ needs to be irradiated with UV radiation to have a photocatalytic activity. UV radiation constitutes about 5% of the total electromagnetic radiation output from sunlight, limiting the practical applications of ZrO_2_.

To improve the photocatalytic performance of ZrO_2_, coupling with other semiconductor oxides has been shown to be an effective approach. The coupling produces mixed oxide semiconductors (MOS) that are capable of efficient charge separation due to the transition of electrons and holes generated from one semiconductor to another. CuO is often the semiconductor oxide of choice because it is a p-type semiconductor, has a small band gap of about 1.2 eV, is cheap and is environmentally friendly [5]. Renuka, L., et al., reported the synthesis of ZrO_2_/CuO materials with excellent photocatalytic activity under visible light by a simple combustion method. The photocatalytic activity of ZrO_2_/CuO obtained was 1.5 times higher than that of commercial P25 [6]. Nanda, B., et al., synthesized mesoporous CuO/ZrO_2_–MCM–41 nanocomposites with high photocatalytic activity, the photoreduction of Cr(VI) to Cr(III) had a 99% degradation efficiency in 30 min [7]. The synergistic effect between ZrO_2_, CuO and TiO_2_ oxides was also studied by Guerrero-Araque and Diana, et al. Co-catalysts are widely used to promote photocatalytic hydrogen production, especially CuO, which has shown a significant improvement in reaction rates [8].

Another use of low-photocatalytic activity is the electron-hole recombination process that occurs in the semiconductor oxide. In photocatalysis, the photocatalytic activity depends on the ability of the catalyst to create electron-hole pairs. Such an electron-hole pair is called an exciton. The excited electron and hole can recombine and release heat. Such exciton recombination leads to a decrease in photocatalytic activity. To reduce the recombination of electrons and holes, it is common to dope transition metal elements, especially rare earth, to prolongation of the exciton lifetime [9].

Recently, Piña-Pérez and Yanet, et al., doped Ce^3+^/Ce^4+^ into Al_2_O_3_, showing significantly improved electron-hole pair separation efficiency. This catalyst was chosen for the photodegradation of other phenolic derivatives such as 4–chlorophenol, p–cresol and 4–nitrophenol. Ce^3+^/Ce^4+^-doped Al_2_O_3_ synthesized by the sol-gel method showed better photocatalytic activity (TiO_2_, P25 Degussa) [10]. The redox pair of Ce^4+^/Ce^3+^-doped α-β phase of Bi_2_O_3_ was reported by Akshatha, S., et al. The Ce^4+^/Ce^3+^-doped Bi_2_O_3_ created a synergistic effect to enhance the photocatalytic activity to degrade Alizarin red S dye [11].

To the best of our knowledge, there are many studies on the doping of Ce^3+^/Ce^4+^ ions into oxide semiconductors for photocatalysis. However, the doping of Ce^3+^/Ce^4+^ ions into mixed semiconductor oxide is a topic that has not been fully investigated. In this paper, we use the hydrothermal method to prepare Ce^3+^/Ce^4+^-doped ZrO_2_/CuO. The Ce^3+^/Ce^4+^-doped ZrO_2_/CuO mixed oxide semiconductor was used for the photocatalytic degradation of methylene blue (MB). X-ray diffraction (XRD), field emission scanning electron microscopy (FE-SEM), transmission electron microscopy (TEM), infrared spectroscopy (FT-IR), energy dispersive X-ray spectroscopy, spectrophotometry UV-Vis and Brunauer–Emmett–Teller (BET) were studied.

## 2. Materials and Methods

### 2.1. Preparation of the Ce^3+^/Ce^4+^-Doped ZrO_2_/CuO Nanocomposites

The Ce^3+^/Ce^4+^-doped ZrO_2_/CuO nanocomposites were synthesized by the hydrothermal method. All chemicals were purchased from Merck (purity > 99%) and were used without further treatment: ZrCl_4_, Cu(NO_3_)_2_.6H_2_O, Ce(SO_4_)_2_.4H_2_O, NaOH, C_2_H_5_OH, Methylene blue.

In a typical experiment, the molar ratio of ZrO_2_/CuO was taken as 1:1, 10 mmol of ZrCl_4_ and 10 mmol of Cu(NO_3_)_2_ were dissolved in 50 mL of deionized water, stirred in a magnetic stirrer at room temperature for 30 min. Next, 0–0.8 mmol Ce(SO_4_)_2_ was added to the above solution. The mixture is stirred vigorously to obtain a clear and homogeneous mixture. After stirring these mixtures for 30 min, they were added to 10 mL of 2 M NH_3_ aqueous solution to gel.

The gel was then transferred into a Teflon-lined 100 mL stainless steel autoclave, which was heated in an oven at 200 °C for 12 h (heated with a Nabertherm furnace, heating rate 5 °C/min). For natural cooling, the entire solution was centrifuged to collect the precipitate and washed with deionized water, and then dried at 80 °C. Pure ZrO_2_ and CuO were prepared with the same procedure. Finally, all the prepared powders were calcined at 600 °C for 2 h and then allowed to cool to room temperature for further experiments.

### 2.2. Characterization

The morphology of the Ce^3+^/Ce^4+^-doped ZrO_2_/CuO nanocomposites was evaluated by using a field emission scanning electronic microscope (JEOL, JSM–7600F, JEOL Techniques, Tokyo, Japan). The surface morphology and microstructure of the nanocomposites were characterized by transmission electron microscopy (TEM) made with a (JEOL, JEM 1010, JEOL Techniques, Tokyo, Japan) operating at 200 kV. For the TEM analyses, the powders were dispersed in ethanol by sonication for 5 min.

FT-IR was performed in the range of 4000–400 cm^−1^ with the help of (FT-IR using a Perkin–Elmer Spectrum BX spectrometer (PerkinElmer Inc., Wellesley, MA, USA) using KBr pellets to identify the functional groups present in the sample. EDS (Hitachi TM4000Plus Tabletop Microscope(Hitachi High-Tech Corporation, Tokyo, Japan)) was the determining element in the composites. The UV-Vis absorption spectrum was recorded using a (UV–1700 PharmaSpec, Shimadzu, Kyoto, Japan) UV-Visible spectrophotometer. The X-ray powder diffraction patterns were characterized using (XRD, D8 Advance, Bruker, Germany) a diffractometer at room temperature (Cu-Kα radiation) with a nickel filter at a scan rate of 2°/min.

### 2.3. Dye Photodegradation

The photocatalytic activity of Ce^3+^/Ce^4+^-doped ZrO_2_/CuO nanocomposites was evaluated by investigating the degradation of methylene blue (MB). Using 30 mL of the MB solution with a dye concentration of 10 mgL^−1^, which was illuminated under a light-emitting 300W Xenon lamp (sunlight simulation) at irradiance (1 Wcm^−2^). Light filters were used to remove wavelengths λ < 420 nm. The distance from the lamp to the MB solution was 10 cm. To limit the influence of external light, the entire experimental system was placed in a dark chamber. The nanocomposite powder loaded into the MB solution was 20 mg. Then the suspension was stirred for 60 min in the dark to obtain balance adsorption and desorption equilibration of the system. About 3 mL was taken from the suspension at different irradiation times, it was then centrifuged at 4000 rpm for 10 min, and degradation measurements were performed by a UV-Vis spectrophotometer. The photodegradation efficiency was calculated using Equation (1):

(1) $$\%H=\frac{C_{0}-C_{t}}{C_{0}}.100$$

where *%H* is the photodegradation efficiency, *C_0_* and *C_t_* are the concentrations of MB at time 0 and *t* (min), respectively.

## 3. Results

### 3.1. X-ray Diffraction

**Figure 1:**
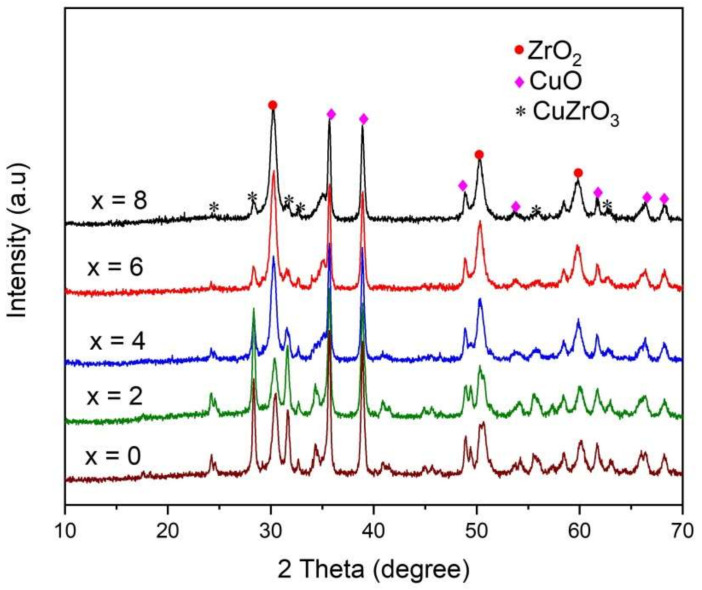
XRD patterns of the ZrO_2_/CuO nanocomposite powder with doping of Ce^3+^/Ce^4+^ (0–8 mol%).

The XRD pattern of the Ce^3+^/Ce^4+^-doped ZrO_2_/CuO nanocomposites is shown in Figure 1. All the peaks in the XRD pattern are well indexed and have all crystallized as multiphase at Ce^3+^/Ce^4+^ different doping concentrations. ZrO_2_/CuO and Ce^3+^/Ce^4+^-doped ZrO_2_/CuO exists in three phases, namely tetragonal ZrO_2_, monoclinic CuO and orthorhombic CuZrO_3_.

The diffraction peaks originating at 2θ = ~ 30.48°, 34.35°, 50.30°, 50.65° and 60.09° correspond to (101), (200), (112), (220) and (202) planes of the tetragonal phase of ZrO_2_ (JCPDS No. 00–050–1089), respectively. The diffraction peaks observed at 2θ = ~ 35.68°, 38.89°, 48.93°, 53.62°, 61.70°, 65.97° and 68.20° are associated with (002), (111), (−202), (020), (−113), (022) and (113) planes, signifying the monoclinic phase of CuO (JCPDS No. 48–1548).

We also observed diffraction peaks at 2θ = ~ 24.33°, 28.29°, 34.34°, 55.56° and 61.72° corresponded to (012), (112), (013), (313) and (402) planes of CuZrO_3_ (PDF Card No. 00–043–0953), respectively. CuZrO_3_ can be formed in a hydrothermal process, where the reaction between ZrO_2_ and CuO occurs according to the chemical equation:

(2) $${{\mathrm{ZrO}}_{2}}_{\;}+\mathrm{CuO}\;\overset{t^{0}}{\to }{\mathrm{CuZrO}}_{3}$$

It is interesting that when the Ce^3+^/Ce^4+^ doping concentration changes, the ratio between the phases changes. In the absence of Ce^3+^/Ce^4+^ doping, the material exists in all three phases, including tetragonal ZrO_2_, monoclinic CuO and orthorhombic CuZrO_3_. However, when increasing the doping concentration of Ce^3+^/Ce^4+^, the diffraction intensity of the CuZrO_3_ phase decreased. According to the authors Dean, James, et al., reported that CuZrO_3_ has a perovskite structure; this structure is less stable due to its low energy, which has a thermodynamic preference to decompose into CuO and ZrO_2_ [12]. We believe that increasing the concentration of Ce^3+^/Ce^4+^ doping increases the ability of phase segregation to CuO and ZrO_2_ is preferred.

### 3.2. Microstructure and Morphology of ZrO_2_/CuO-Doped Ce^3+^/Ce^4+^ Nanocomposites

**Figure 2:**
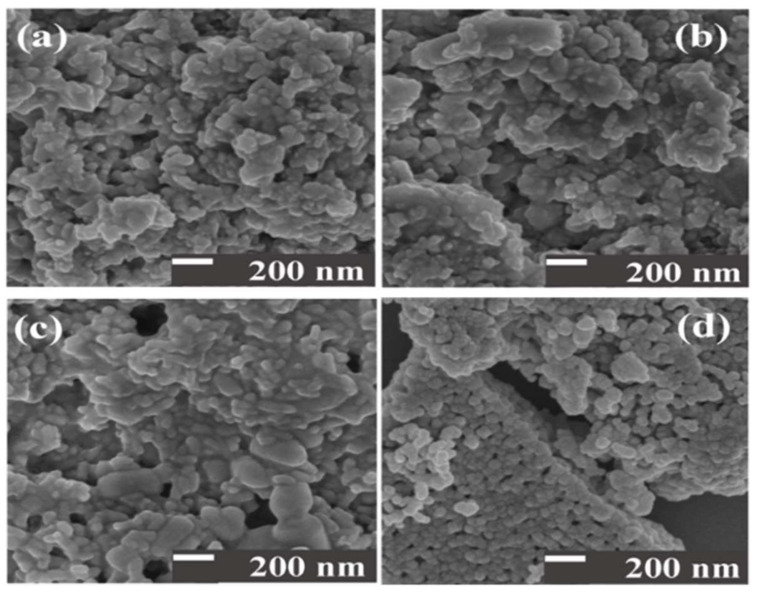
FE-SEM of x mol% Ce^3+^/Ce^4+^-doped ZrO_2_/CuO: (**a**) x = 2; (**b**) x = 4; (**c**) x = 6; (**d**) x = 8.

The microstructure of the Ce^3+^/Ce^4+^-doped ZrO_2_/CuO synthesized by the hydrothermal method was analyzed by FE-SEM. Figure 2a–d show that the Ce^3+^/Ce^4+^-doped ZrO_2_/CuO catalysts are spheres with non-uniform size distribution. When the Ce^3+^/Ce^4+^ doping concentration changed from 2 mol% to 8 mol%, the morphology of the catalysts was not significantly affected.

The size of the catalyst, as well as the surface area, greatly influence the photocatalytic efficiency. The small size of the material shows that the ability to adsorb the MB on the surface will be better, creating conditions for the oxidizing radicals to easily react with the colourant.

To better understand the shape, size and crystal formation of the Ce^3+^/Ce^4+^-doped ZrO_2_/CuO catalyst, we studied the transmission electron microscopy (TEM) images.

**Figure 3:**
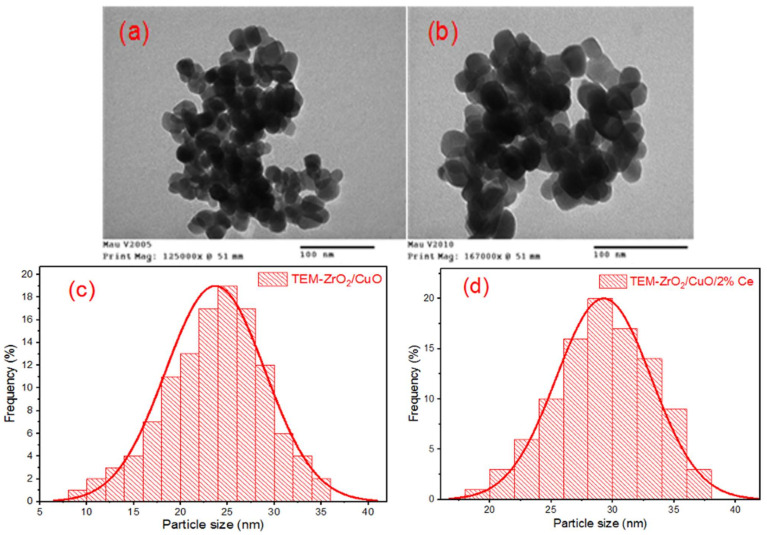
TEM and the particle size distribution curve of ZrO_2_/CuO (**a**,**c**) and 2 mol% Ce^3+^/Ce^4+^-doped ZrO_2_/CuO (**b**,**d**).

Figure 3 shows that the particle size of the Ce^3+^/Ce^4+^-doped ZrO_2_/CuO catalyst is larger than that of ZrO_2_/CuO. According to the particle size distribution curves in Figure 3c–d, ZrO_2_/CuO concentrates around 20 nm, while Ce^3+^/Ce^4+^-doped ZrO_2_/CuO concentrates around 30 nm.

### 3.3. Fourier Transform Infrared Spectroscopy and Energy Dispersive X-ray Spectroscopy of the Ce^3+^/Ce^4+^-Doped ZrO_2_/CuO Nanocomposites

**Figure 4:**
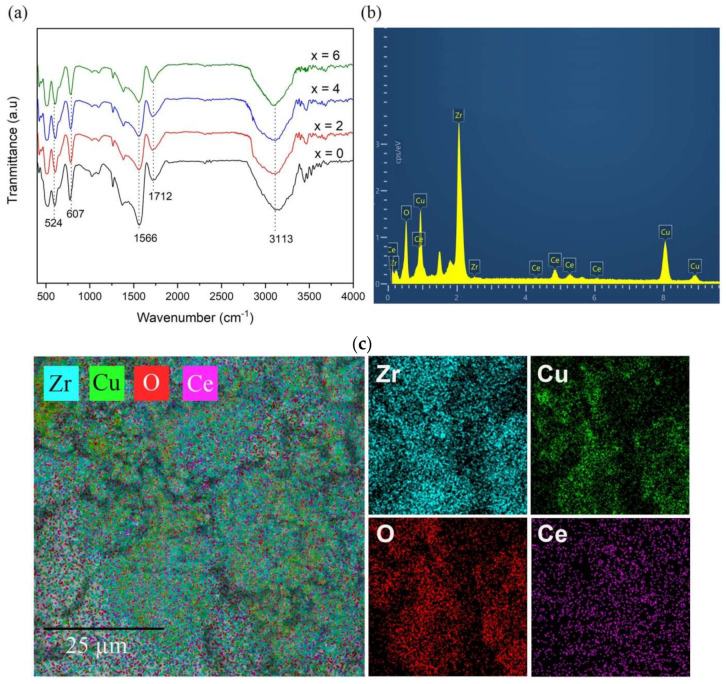
(**a**) FT-IR of x mol% Ce^3+^/Ce^4+^-doped ZrO_2_/CuO (x = 0; 2; 4; 6), (**b**) EDS, (**c**) mapping imaging of 8 mol% Ce^3+^/Ce^4+^-doped ZrO_2_/CuO.

Figure 4 shows the FT-IR spectra of ZrO_2_/CuO and Ce^3+^/Ce^4+^-doped ZrO_2_/CuO. The Ce^3+^/Ce^4+^-doped ZrO_2_/CuO materials all have absorption maximums around 3113–3153 cm^−1^, which is typical for the –OH group of wet water. The absorption peaks at 1710–1712 cm^−1^ and 1556–1566 cm^−1^ are characteristic of the vibrations of the Zr–O–H group in the material. In addition, the materials have an absorption maximum of 601–607 cm^−1^, which can be attributed to the vibrational stretching mode of the Cu-O bond. Bands at 424–524 cm^−1^ can be assigned to the Zr–O vibration mode in composites.

Figure 4b shows the presence of elements Ce, Zr, Cu and O, confirming the presence of Ce doping in ZrO_2_/CuO nanocomposites. We performed EDS mapping, which is equipped with SEM. Figure 4c clearly show the uniform spread of Zr, Cu, O and Ce elements.

### 3.4. Nitrogen Adsorption-Desorption Isotherms of the Ce^3+^/Ce^4+^-Doped ZrO_2_/CuO Nanocomposites

**Figure 5:**
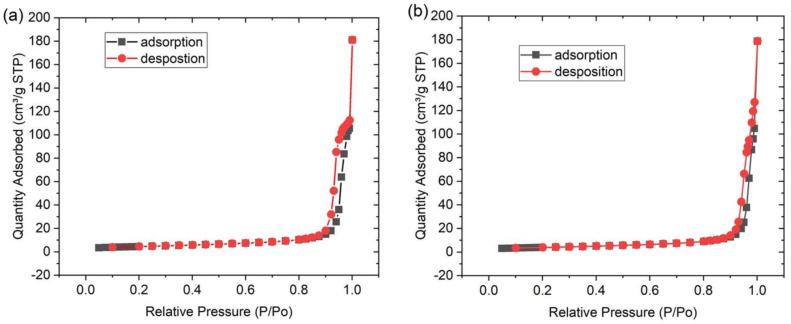
Nitrogen adsorption-desorption isotherms of (**a**) ZrO_2_/CuO, and (**b**) 2 mol% Ce^3+^/Ce^4+^-doped ZrO_2_/CuO.

The specific surface areas of the Ce^3+^/Ce^4+^-doped ZrO_2_/CuO and ZrO_2_/CuO nanocomposites were determined by measuring the nitrogen adsorption-desorption isotherms, as shown in Figure 5. All of them are all type III adsorption isotherms [13]. The BET surface areas of ZrO_2_/CuO and Ce^3+^/Ce^4+^-doped ZrO_2_/CuO are 15.59 and 13.52 m^2^g^−1^. The large surface area of the catalyst facilitates the adsorption of the pigment to the surface of the material, thereby improving the photocatalyst efficiency.

### 3.5. UV-Vis Absorption Spectrum of Ce^3+^/Ce^4+^-Doped ZrO_2_/CuO Nanocomposites

**Figure 6:**
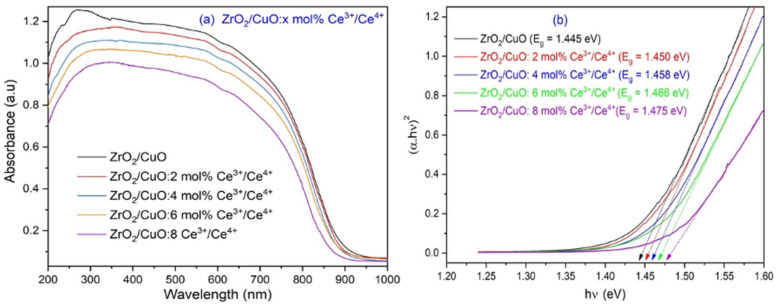
(**a**) The UV-Vis DRS and (**b**) The Kubelka-Munk energy curve versus the band gap (eV) of the x mol% Ce^3+^/Ce^4+^-doped ZrO_2_/CuO (x = 0; 2; 4; 6; 8).

Figure 6 shows the UV-Vis absorption spectrum and the bandgap energy of the Ce^3+^/Ce^4+^-doped ZrO_2_/CuO. The results show that the Ce^3+^/Ce^4+^-doped ZrO_2_/CuO oxide composite material has an absorption region from UV to the visible region (200–1000 nm). To calculate the band gap, the Tauc relation is used:

αhν = A(hν − E_g_)^n^ (3)

where α is the absorption coefficient, h is the Planck constant, ν is the frequency of the incident photon, A is a constant that depends on the transition probability, E_g_ is the band gap energy and n is an index that depends on the transition probability of the nature of the electronic transition.

The bandgap energy of Ce^3+^/Ce^4+^-doped ZrO_2_/CuO was found to be in the range of 1.445–1.475 eV. When increasing the Ce^3+^/Ce^4+^ doping concentration from 0 to 8 mol%, the band gap increased slightly from 1.445 to 1.475 eV. When Ce was doped into ZrO_2_/CuO, the 5d and 6s orbitals of Ce overlapping with the d orbitals of Cu and Zr can increase the size of the valence band and also lower the position of its maximum, thereby increasing the band gap of mixed semiconductor oxide. The increase in the optical band gap upon Ce doping is similar to the increase in the band gap of Ag_2_O upon Zn doping, as reported by De, Arup Kumar et al. [14]. The decrease in the band gap of the ZrO_2_/CuO nanocomposites material compared with that of pure ZrO_2_ is mainly due to the introduction of CuO into the ZrO_2_ host matrix. It was found that when two metals combine, the band gap decreases and thus a shift to the visible region is observed [15].

The narrowing of the band gap can be attributed to the appearance of an impurity region (CuO) formed by overlapping impurity states and local states formed by the combination of Cu 2p and Zr 3p [7]. This result is similar to previous studies [16]. The photocatalytic reaction is initiated by the absorption of light with an energy equal to or greater than the band gap of the semiconductor. The band gap is in the range of 1.445–1.475 eV, so it is expected that the catalyst material can absorb visible light.

### 3.6. Photocatalytic Activity of Ce^3+^/Ce^4+^-Doped ZrO_2_/CuO Nanocomposites

**Figure 7:**
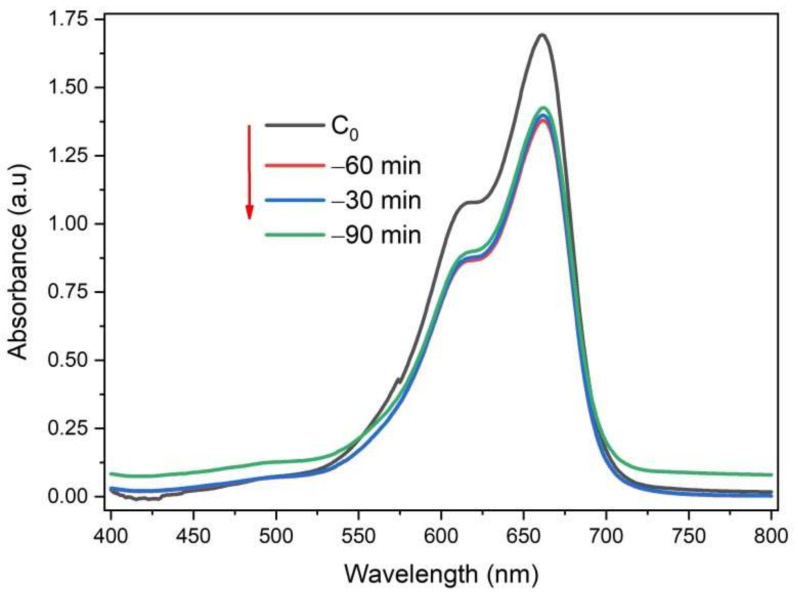
UV-Vis spectrum of MB after being adsorbed by ZrO_2_/CuO-doped 8 mol% Ce^3+^/Ce^4+^.

To investigate the adsorption balance, 20 mg of 8 mol% Ce^3+^/Ce^4+^-doped ZrO_2_/CuO was added into 30 mL of 10 mgL^−1^ MB solution. The suspension was stirred in the dark; samples were measured via UV-Vis spectroscopy. Figure 7 shows that after the first 30 min, the concentration of MB strongly decreased, then at 60 min slightly decreased, and at 90 min, the concentration of MB increased slightly (desorption). It is shown that after 60 min of adsorption, equilibration has occurred.

**Figure 8:**
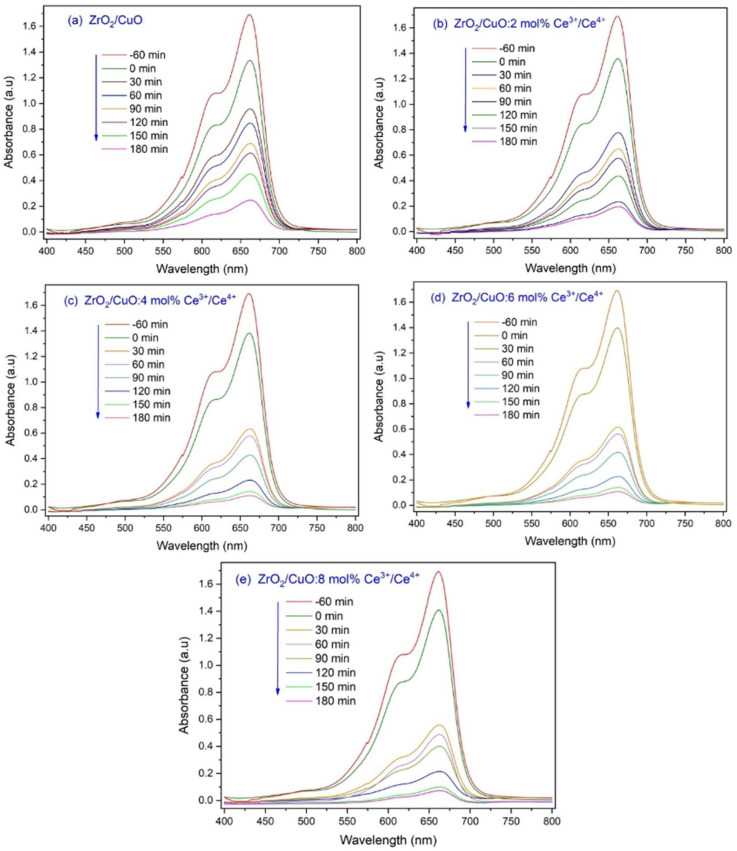
UV-Vis absorbance of MB under visible light at different times in the presence of x mol% Ce^3+^/Ce^4+^-doped ZrO_2_/CuO: (**a**) x = 0; (**b**) x = 2; (**c**) x = 4; (**d**) x = 6; (**e**) x = 8.

Figure 8 shows the UV-Vis absorption spectrum of MB under visible light at different times in the presence of Ce^3+^/Ce^4+^-doped ZrO_2_/CuO. The evolution of MB photodegradation was monitored by measuring the absorbance at the wavelength of about λ = 662 nm at different times. The maximum absorption peaks of MB gradually decreased and almost disappeared in 180 min of illumination.

The reactions that occur during photocatalysis are described below. The reaction begins by generating excitons on the metal oxide surface (MeO) [17]:

MeO + hν → MeO (h^+^ + e^−^)

Oxidation reactions due to the photocatalytic effect:

h ^+^ + H_2_O → H^+^ + •OH

2h^+^ + 2 H_2_O → 2H^+^ + H_2_O_2_

H_2_O_2_ → 2 •OH

Reduction reaction due to the photocatalytic effect:

e^−^ + O_2_ → •O_2_^−^

•O_2_^−^ + H_2_O + H+ → H_2_O_2_ + O_2_

H_2_O_2_ → 2•OH

•OH + Dye → Degradation

•O_2_^−^ + Dye → Degradation

**Figure 9:**
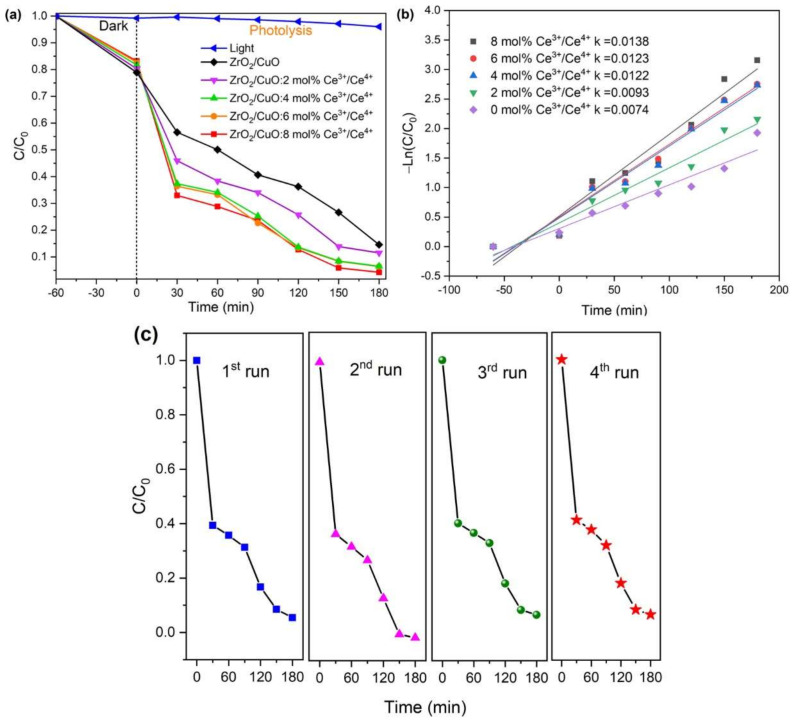
(**a**) Photodegradation of MB, (**b**) the reaction rate constant k of the MB degradation in the presence of x mol% Ce^3+^/Ce^4+^doped ZrO_2_/CuO (x = 0; 2; 4; 6; 8), (**c**) reuse of 8 mol% of Ce^3+^/Ce^4+^ doping ZrO_2_/CuO.

Figure 9a shows the ZrO_2_/CuO catalyst, the photocatalytic efficiency of MB decomposition by Ce^3+^/Ce^4+^-doped ZrO_2_/CuO increased significantly. The Ce^3+^/Ce^4+^ doping concentration increased, and the photocatalytic efficiency under visible light also increased. Although the E_g_ values of ZrO_2_/CuO and ZrO_2_/CuO-doped Ce^3+^/Ce^4+^ were not significantly different, the results showed that the photocatalytic degradation efficiency was enhanced by Ce^3+^/Ce^4+^ doping. This could be due to the fact that as the Ce^3+^/Ce^4+^ ion concentration increases, an increase in oxygen vacancy defects in the crystal lattice occurs. This leads to the redox pair Ce^3+^/Ce^4+^ being capable of shifting between Ce_2_O_3_ and CeO_2_, which reduces electron-hole recombination [18].

The MB degradation processes all show first-order kinetics by plotting ln(*C/C_0_*) versus irradiation time, *t*. The apparent response rate constant (k_app_) was calculated from the slope of the curve, as illustrated in Figure 9b.

The k_app_ value continuously increased as the Ce^3+^/Ce^4+^ doping concentration increased, with x = 0, k_app_ = 0.0074 min^−1^; x = 2, k_app_ = 0.0093 min^−1^; x = 4, k_app_ = 0.0122 min^−1^; x = 6, k_app_ = 0.0123 min^−1^; and x = 8, k_app_ = 0.0138 min^−1^. It was shown that 8 mol% Ce^3+^/Ce^4+^-doped ZrO_2_/CuO exhibited the highest k_app_ = 0.0138 min^−1^, much higher than that of undoped ZrO_2_/CuO with Ce^3+^/Ce^4+^.

Figure 9c shows the stability of the Ce^3+^/Ce^4+^-doped ZrO_2_/CuO nanocomposites. The stability of the MOS material was evaluated by performing photocatalyst recycling experiments for a duration of 180 min. The activity was found to be roughly the same in the two repeated runs, and then there was a slight decrease in the third and fourth runs.

We assume that there is metal-to-metal charge transfer (MMCT). The MMCT effect is assumed to be: Zr^4+^–O–Ce^4+^ to Zr^4+^–O–Ce^3+^ and Zr^4+^–O–Cu^2+^ to Zr^4+^–O–Cu^+^. The effect is when two different metals in the mixture form an oxygen bridge, which helps electrons and holes move efficiently and avoid recombination, increasing the photocatalytic efficiency of the Ce^3+^/Ce^4+^-doped ZrO_2_/CuO catalyst [19]. Furthermore, Ce^3+^/Ce^4+^ ions act as electron-trapping sites reducing charge-pair recombination and thus enhancing photocatalytic activity [20,21].

Table 1 shows the photocatalytic degradation of MB using the various heterojunction photocatalyst. It shows that the combination of semiconductor oxides, or metal ion doping, shows an improved rate constant compared to single semiconductor oxides.

**Figure 10:**
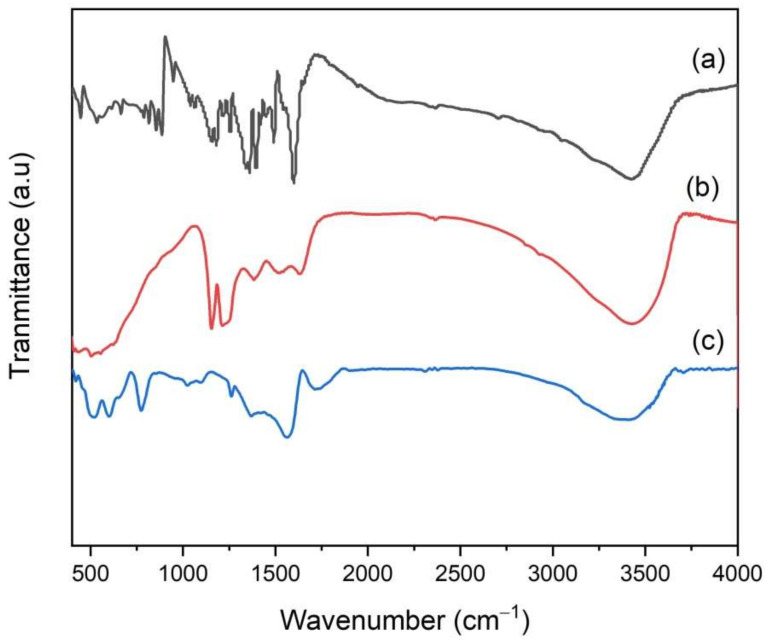
FT-IR spectrum of (a) MB, (b) MB adsorbed onto Ce^3+^/Ce^4+^-doped ZrO_2_/CuO before irradiation, (c) MB adsorbed on Ce^3+^/Ce^4+^-doped ZrO_2_/CuO 180 min after irradiation.

The ZrO_2_/CuO-doped Ce^3+^/Ce^4+^ photocatalyst can be reproducible and without destruction. This reproducible process can be confirmed by FT-IR, as shown in Figure 10. It is clear that the characteristic bands of MB, which are at 1383, 1324, 1245, 884 and 670 cm^−1^, appear after adsorbing [31], while they disappear after 180 min of irradiation. Combined with the results of photocatalytic reuse experiments, it is proven that the Ce^3+^/Ce^4+^-doped ZrO_2_/CuO nanocomposites have stability.

## 4. Conclusions

Ce^3+^/Ce^4+^-doped ZrO_2_/CuO nanocomposites have been synthesized by the simple hydrothermal method. The obtained material is a mixed semiconductor oxide ZrO_2_ and CuO. The coupling between CuO and ZrO_2_ oxides has shown that the light absorption capacity is extended from UV to the visible region. Photocatalytic activity is enhanced when doping Ce^3+^/Ce^4+^ ions because Ce^3+^/Ce^4+^ ions have higher separation efficiency of charge carriers, which reduces electron-hole recombination. The Ce^3+^/Ce^4+^-doped ZrO_2_/CuO nanocomposites showed significantly improved photocatalytic activity. High photocatalysis activity with 94.5% degradation efficiency of MB was achieved after 180 min under visible light irradiation. It is believed that these materials will have promising applications in the wastewater treatment field.

## Figures and Tables

**Table 1:** Photocatalytic degradation of MB dye using the various heterojunction photocatalyst.

Photocatalyst	Synthesis Method	Light Source	Rate Constant (min^−1^)	Reference
Fe_2_O_3_/graphene/CuO	Solvothermal	Visible	72.5 × 10^−3^	[22]
CuO/ZnO	Impregnation	UV lamp	182 × 10^−3^	[23]
ZrO_2_/AgCl:Eu^3+^	Sol-gel	Visible	14 × 10^−3^	[24]
C-doped ZrO_2_	Sol-gel	UVC lamp	7.3 × 10^−3^	[25]
N-TiO_2_/ZrO_2_	Hydrothermal	UV light	29 × 10^−3^	[26]
ZrO_2_/CuO	Combustion	Visible	11.19 × 10^−3^	[6]
GO-ZrO_2_	Co-precipitation	Visible	57.5 × 10^−3^	[27]
CeO_2_	Precipitation	UV light	12.1 × 10^−3^	[28]
V_2_O_5_-CeO_2_	Precipitation	Visible	108 × 10^−3^	[29]
CeO_2_/TiO_2_	Precipitation	Visible	34 × 10^−3^	[30]
Ce^3+^/Ce^4+^-doped ZrO_2_/CuO	Hydrothermal	Visible	13.8 × 10^−3^	This work

## Data Availability

All the data are available within the manuscript.
